# Targeting growth plate senescence: a novel adjunct to GH therapy in pediatric osteosarcoma survivors

**DOI:** 10.1097/MS9.0000000000004289

**Published:** 2025-11-11

**Authors:** Mashal Naveed, Mawra Naveed, Muhammad Talha, Mahnoor Fatima, Muhammad Hanan

**Affiliations:** a Department of Medicine, Allama Iqbal Medical College, Lahore, Pakistan; b Department of Medicine, Shaikh Khalifa Bin Zayed Al-Nahyan Medical and Dental College, Lahore, Pakistan; c Department of Medicine, King Edward Medical University, Lahore, Pakistan; d Department of Medicine, Shaheed Ziaur Rahman Medical College, Rajshahi Medical University, Bogura Bangladesh

*To the Editor,*

Pediatric osteosarcoma, the most prevalent primary malignant bone neoplasm, originates from mesenchymal osteogenic progenitor cells during rapid skeletal growth and constitutes about 3–6% of all pediatric malignancies. Symptoms include chronic bone pain, restricted joint mobility, swelling, and advanced-stage fractures^[1]^. Long-term survivors often demonstrate Growth Hormone Deficiency (GHD) attributable to damage to the hypothalamic-pituitary axis, leading to short stature^[2]^. Although growth hormone (GH) replacement therapy can restore circulating GH levels, it does not reverse growth plate senescence. This letter emphasizes the potential of antisenescence therapy as an adjunct to GH, specifically targeting senescent chondrocytes to maintain growth potential. This is in line with the TITAN Guidelines on the need for transparency in AI use in health care^[3]^.

Growth plate chondrocyte senescence is a physiological process in which the chondrocytes undergo hypertrophic proliferation and then apoptosis, leading to the closure of the epiphyses and the attainment of adult height. In the past, this process was viewed as a barrier to growth; however, now this concept is being used in therapeutics^[4]^. Using anti-senescence therapy, such as senolytics, which are drugs that kill senescent cells by targeting specific senescent cell anti-apoptotic pathways or senomorphics, which are drugs that modify the behavior of senescent cells by suppressing the senescence-associated secretory phenotype, may offer significant potential in overcoming the adverse effects of GHD in affected children^[5]^.

Conventionally, pediatric osteosarcoma with GHD was treated with GH replacement therapy, which showed variable effectiveness and did not specifically target the senescent cells of the growth plate^[6]^. In contrast to this, anti-senescence therapies could target the senescent cells directly, delaying epiphyseal fusion and hence restoring growth potential^[4,5]^. Furthermore, combining senescence-focused strategies with endocrine therapy may offer dual therape utic benefit^[4,7]^. This integrated approach may ensure better treatment for the survivors of pediatric osteosarcoma with GHD.

Though promising, anti-senescence therapy faces substantial limitations. First, although it has been tested in lab and animal studies, direct clinical validation in pediatric osteosarcoma survivors is still lacking. Second, senescence is a natural phenomenon, so interfering with it may result in severe complications, as disturbed growth plate biology, skeletal abnormalities, or even tumorigenic effects. Moreover, a recent study by Carpenter *et al* shows how anti-senescence therapy is highly heterogeneous, so the chondrocyte response to it may vary substantially^[8]^. Furthermore, difference in treatment regimens, age, and genetic makeup makes it difficult to translate these findings into real clinical practice.

In conclusion, promising results can be obtained by the use of anti-senescent drug therapy to delay the senescence by selectively targeting the chondrocytes of the growth plate in this clinical subgroup. However, this strategy receives poor support from clinical trials and is at its initial stage. Furthermore, there is a need for more clinical trials on this strategy about its safety, efficacy, tolerability, and side effects, and it is essential for the approval of its use in clinical settings.

## Data Availability

Not applicable to this study.
